# Application and translational research of magnesium in nursing care of orthopedic diseases: from mechanism to clinical translation

**DOI:** 10.3389/fchem.2026.1817443

**Published:** 2026-04-17

**Authors:** Shaoying Sheng, Chengfei Yu, Manman Wang, Yiqing Tao, Yaojing Ma

**Affiliations:** 1 Nursing Department, the Second Affiliated Hospital, Zhejiang University School of Medicine, Hangzhou, Zhejiang, China; 2 Department of Orthopedics, The Second Affiliated Hospital, Zhejiang University School of Medicine, Hangzhou, Zhejiang, China

**Keywords:** biomaterial, magnesium, nursing care, orthopedic diseases, translational medicine

## Abstract

Magnesium is an essential bioactive ion that plays a central role in skeletal physiology. However, its contributions to bone biology and orthopedic practice are only beginning. Beyond its structural incorporation into bone mineral, magnesium dynamically regulates osteoblast and osteoclast function through signaling networks, including PI3K/Akt, TRPM, and the OPG/RANKL/RANK axis. Disruption of magnesium homeostasis has been increasingly implicated in osteoporosis, delayed fracture healing, and other skeletal pathologies. Concurrently, the emergence of biodegradable magnesium-based biomaterials has introduced new opportunities for next-generation orthopedic implants that actively promote bone regeneration while eliminating the need for implant removal. Clinical implementation in magnesiumsupplementation is also growing, with evidence suggesting benefits for bone health, postoperative recovery, and pain management. This review summarizes the physiological functions of magnesium, magnesium related signals in bone metabolism, magnesium-related skeletal diseases, clinical applications of magnesium alloys, and nursing strategies for orthopedic patients.

## 1 Introduction

Magnesium is the fourth most abundant cation in human body and plays a crucial role in maintaining physiological homeostasis. Approximately 50%–60% of total body magnesium is stored in bones, where it participates in regulating osteoblast and osteoclast activities. As an essential factor for numerous enzymes, magnesium is involved in DNA and RNA synthesis, protein metabolism, and ATP-dependent reactions, highlighting its fundamental role in cellular metabolism (Jallot, et al., 2005). Accumulating evidence indicates that magnesium regulates bone metabolism through multiple signaling pathways, including PI3K/Akt, OPG/RANKL/RANK, TRPM, etc. These pathways coordinate osteoblast proliferation and differentiation, osteoclast-mediated bone resorption, thereby maintaining bone homeostasis. Dysregulated magnesium metabolism has been associated with osteoporosis, impaired fracture healing, and other skeletal disorders (Xia, et al., 2025).

**FIGURE 1:**
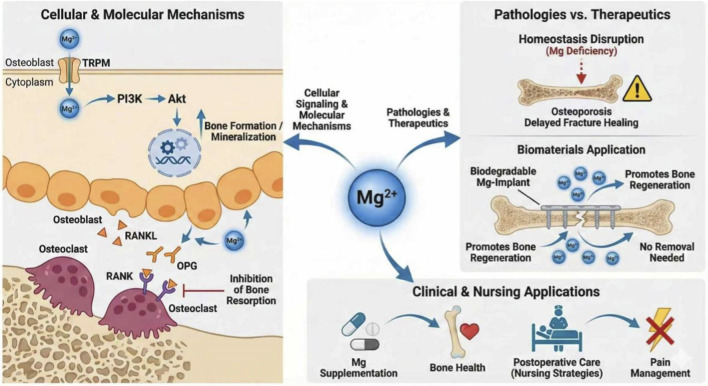
Overview of magnesium’s role in bone metabolism. Schematic overview of magnesium (Mg^2+^) in bone metabolism and clinical applications. Mg^2+^ enters osteoblasts via TRPM channels, activating the PI3K/Akt pathway to promote bone formation and mineralization, while inhibiting osteoclast activity through modulation of the RANK/RANKL/OPG axis. Mg deficiency disrupts bone homeostasis, leading to osteoporosis and delayed fracture healing, whereas biodegradable Mg implants support bone regeneration. Clinically, Mg supplementation contributes to bone health, postoperative care, and pain management.

In parallel, magnesium-based biomaterials have attracted increasing attention as biodegradable orthopedic implants due to their favorable mechanical properties, biocompatibility, and osteo-inductive potential. However, existing reviews mostly focus on the molecular biological mechanisms or material science research of magnesium, while few systematically integrate the basic mechanism of magnesium regulating bone metabolism, clinical translation of magnesium-based biomaterials, and full-cycle nursing management strategies for orthopedic patients, leading to a disconnection between basic research and clinical nursing practice. Meanwhile, the molecular mechanisms of magnesium-mediated bone regulation and its standardized clinical nursing management system remain incompletely understood (Alexander, et al., 2008). This review summarizes the physiological role of magnesium, magnesium-regulated bone signals, magnesium-related skeletal diseases, and clinical and nursing managements, aiming to provide a translational perspective for orthopedic research and practice. This review is the first to systematically elaborate the translational application of magnesium from basic molecular mechanisms to orthopedic clinical nursing practice, fills the gap in the interdisciplinary field of orthopedic nursing and translational biomedicine, summarizes the physiological role of magnesium, magnesium-regulated bone signals, magnesium-related skeletal diseases, clinical application of magnesium alloys, and full-process nursing management strategies for orthopedic patients, aiming to provide evidence-based theoretical guidance and practical reference for orthopedic clinical research and nursing practice (Figure 1).

## 2 Magnesium physiology and metabolism

Available data indicate that the total amount of magnesium in the adult human body is approximately 24–28 g, and the recommended daily dietary intake for healthy adults is 310–420 mg (Gröber, et al., 2015). Magnesium is abundant in green vegetables, followed by legumes, cereals, fish, meat, eggs, and dairy products; therefore, the magnesium intake in daily diets is generally sufficient to meet physiological requirements (Volpe, 2015). Magnesium is mainly absorbed in the small intestine, and the absorption amount is proportional to dietary magnesium intake, with an absorption rate of approximately 30% of the ingested amount (Mooren, 2015). Recent studies have shown that magnesium is actively absorbed in the intestine, and certain amino acids can promote its solubility and increase its absorption; thus, a high-protein diet can enhance magnesium absorption. Magnesium excretion is primarily mediated by the kidneys (Blaine, et al., 2015; Volpe, 2013).

From a physiological perspective, magnesium is an essential element for the human body. Magnesium maintains the structure and function of DNA and serve as cofactors for DNA polymerase and RNA polymerase (Seitz, et al., 2014). Magnesium is involved in almost every step of protein synthesis. In addition, all enzymes related to ATP metabolism require magnesium, such as hexokinase, creatine kinase, and Na^+^/K^+^-ATPase. During oxidative phosphorylation, magnesium plays a critical role (Nielsen and Lukaski, 2006). Once magnesium is absent, mitochondria cannot carry out oxidative phosphorylation (Li, et al., 2022).

Especially, magnesium plays particular roles in the central nervous system, peripheral nerves, and muscles. It has curare-like effects, inhibiting neuromuscular transmission, acetylcholine release, and the sensitivity of motor endplates to acetylcholine. Magnesium also exerts inhibitory effects on the central nervous system (Olinger, 1989). When Mg concentration reaches 10 mg/L, sedative and paralytic effects occur, then motion and deep reflexes disappear (Pickering, et al., 2020).

## 3 Magnesium regulated pathways in bone metabolism

Bone metabolism involves a dynamic balance between osteoblasts and osteoclasts, regulated by hormones, ion channels, and signaling pathways. Bone metabolism is important in a lot of orthopedic diseases, such as osteoporosis, or the subchondral bone in osteoarthritis (Huang, et al., 2021). Recent studies indicate that magnesium-related pathways include PI3K/Akt, OPG/RANKL, TRPM, and Wnt signaling pathways (Table 1).

**TABLE 1:** Effects of abnormal magnesium levels on bone tissue.

Magnesium status	Osteoblast effects	Osteoclast effects	Overall bone tissue changes
Deficiency	Reduced osteogenic markers; inhibited proliferation/differentiation	Increased numbers and resorption activity (low OPG, high RANKL)	Osteoporosis, reduced bone density, delayed fracture healing
Excess (hypermagnesemia)	Inhibited mineral deposition/ALP activity; impaired proliferation/adhesion	Stimulated bone resorption (in animal studies)	Abnormal mineralization, osteomalacia-like manifestations, widened growth plate
Normal	Activated osteogenic pathways; maintained marker expression	Balanced activity (OPG/RANKL equilibrium)	Dynamic bone formation/resorption balance, stable microstructure and bone mass

We have listed the effects of different Magnesium levels on bone tissues, which is the mechanism of clinical intervention strategies.

### 3.1 PI3K/Akt signaling pathway

The PI3K/Akt signaling pathway plays an important role in various cellular biological behaviors, including proliferation, differentiation, and apoptosis. In bone metabolism, this pathway primarily functions through activation of Akt downstream of phosphoinositide 3-kinase (PI3K). Akt, also known as protein kinase B (PKB), further activates downstream target proteins and serves as a key regulator of bone formation and remodeling. Experimental studies have shown that Akt1/Akt2 knockout mice exhibit delayed ossification (Peng, et al., 2003), and Akt1 knockout mice show shortened skeletal length and delayed formation of secondary ossification centers (Ulici, et al., 2009), indicating that Akt and its downstream targets are indispensable components of bone metabolism.

Regulation of purines during osteoblast differentiation is also associated with the PI3K/Akt pathway, mainly through PI3K/Akt-mediated regulation of ATP and UTP, which are closely related to osteoblast proliferation and differentiation. Different concentrations of magnesium ions exert differential effects on the PI3K/Akt pathway in osteoblasts; studies have shown that 6–10 mmol/L magnesium ions upregulate p-Akt expression, whereas 18 mmol/L magnesium ions downregulate its expression. These findings suggest that the osteogenic effects of magnesium alloys may be mediated by magnesium ions released during degradation via activation of the PI3K/Akt pathway. The PI3K/Akt pathway also regulates osteoclast proliferation and differentiation (Liang, et al., 2012; Rangaswami, et al., 2012). Inhibition of PI3K/Akt reduces osteoclast bone resorption activity (Adapala, et al., 2014). Experimental inhibition of PI3K with LY294002 significantly reduced RANKL/CSF-induced osteoclastogenesis, suggesting interaction between the PI3K/Akt and OPG/RANKL pathways (Qiu, et al., 2008). As a central regulatory network linking osteoblast and osteoclast pathways, the PI3K/Akt pathway maintains bone mass and bone turnover balance when activated by appropriate magnesium concentrations.

Magnesium ions activate the PI3K/Akt pathway, leading to changes in bone-related signaling molecules, such as increased expression of alkaline phosphatase and bone morphogenetic proteins, thereby promoting osteoblast proliferation and differentiation (Wu C. M. et al., 2013; Wu S. S. et al., 2013). Appropriate magnesium concentrations activate downstream target proteins by regulating Akt expression, influencing osteoblast proliferation and differentiation (Ghosh-Choudhury, et al., 2002; Guntur and Rosen, 2011; Li F. Y. et al., 2011; McGonnell, et al., 2012). Moreover, the PI3K/Akt pathway regulates bone mass by promoting the differentiation of bone marrow mesenchymal stem cells into osteoblasts. Macrophage growth factor receptors on mesenchymal stem cells mediate proliferation and differentiation via the PI3K/Akt pathway (Hu, et al., 2025). Magnesium ions can also function as intracellular second messengers (Li L. R. et al., 2011). Upon external stimulation, intracellular free magnesium levels increase and regulate mitosis through the “membrane–magnesium–mitosis” model (Günther, 2006). During mitotic signaling, intracellular magnesium concentrations rapidly increase, activating the PI3K pathway and promoting osteoblast mitosis (Rubin, 2007). Interestingly, Zheng et al. found that the same concentration of magnesium ions can regulate Akt pathway in different cells to quite different levels (Zheng, et al., 2024). It was reported that magnesium ions can activate the phosphorylation of Akt in osteoblasts and BMSCs, while it inhibited the process in osteoclasts. The finding is quite important for revealing the mechanism of magnesium in regulating the PI3K/Akt pathway.

### 3.2 OPG/RANKL/RANK signaling pathway

Receptor activator of nuclear factor-κB (RANK) is a type I transmembrane protein of the TNF family, highly expressed on osteoclast precursors, mature osteoclasts, dendritic cells, mammary epithelial cells, and certain cancer cells. RANKL is a type II homotrimeric transmembrane protein. RANKL and macrophage colony-stimulating factor (M-CSF) are essential for initiating gene transcription during osteoclast differentiation and are key factors released by osteoblasts to trigger osteoclast differentiation (Haÿ, et al., 2009; Rangaswami, et al., 2012). Osteoprotegerin (OPG), a member of the TNF receptor family, is considered a final mediator of osteoclastogenesis and function (O'Brien, et al., 2001). Factors that stimulate bone resorption decrease OPG expression. RANKL binds to RANK on osteoclast precursors, promoting osteoclast differentiation and bone resorption. Magnesium ions regulate serum levels of OPG and RANKL, thereby influencing osteoclast biological activity and maintaining bone metabolic balance. OPG inhibits osteoclast differentiation and increases bone density, whereas OPG ligand (OPGL, also known as osteoclast differentiation factor) induces osteoclast formation *in vitro*. RANK is mainly expressed on monocyte–macrophage lineage cells and osteoclast precursors, where RANKL binding promotes osteoclast maturation. Magnesium deficiency decreases OPG and increases RANKL levels, leading to increased osteoclast numbers and bone resorption (Bae and Kim, 2010). M-CSF increases the osteoclast precursor pool, while RANKL promotes differentiation and activation and inhibits apoptosis of osteoclasts (Schoppet, et al., 2002). Osteoblasts also secrete OPG to competitively inhibit RANKL binding to RANK, thereby suppressing osteoclast activation and differentiation (Kulkarni, et al., 2010).

Abnormal magnesium concentrations can regulate osteoblast and osteoclast proliferation and differentiation through the OPG/RANKL/RANK pathway, affecting bone mass. In the previous study, a magnesium degradation-induced variable fixation plat promoted faster and stronger bone healing in rabbits, potentially by accelerating the expression of BMP2 and modulating the OPG/RANKL/RANK signaling axis. The results revealed that the expression levels of BMP2 and OPG increased during early fracture stages but decreased in late fracture stages, whereas RANKL expression showed the opposite trend in the variable fixation group (Wen, et al., 2024). Moreover, ZA-CaP bilayer coating Mg-Sr alloy could regulate the crosstalk of osteoblast-osteoclast through OPG/RANKL/RANK signaling pathway, which promoting the balance of bone remodeling process (Li M. et al., 2019). Meanwhile, it was found that the supplementation of magnesium improves Serum OPG/RANKL in calcium-deficient ovariectomized rats (Bae and Kim, 2010). From these works, we can find that magnesium is linked with the crosstalk between osteoblasts and osteoclasts through OPG/RANKL/RANK pathway, which can further affecting the bone mass.

### 3.3 TRPM

The melastatin-related transient receptor potential (TRPM) channel family consists of eight members (TRPM1–TRPM8) and represents an important class of cation channels in mammalian cell membranes (Sorrentino, et al., 2026). Functional TRPM channels form tetramers, and structural integrity of N- and C-terminal domains is essential for their function. TRPM channels can be activated by various factors, including Ca^2+^ and Mg^2+^.

Among them, TRPM6 and TRPM7 regulate intracellular magnesium homeostasis. TRPM6 mainly maintains magnesium stability (Walder, et al., 2009), whereas TRPM7 regulates magnesium balance. Appropriate extracellular magnesium concentrations induce osteoblast proliferation and migration (Bates-Withers, et al., 2011), and TRPM7 expressed on osteoblasts senses extracellular magnesium fluctuations to regulate physiological activities (Schmitz, et al., 2003). Low magnesium levels activate TRPM7 channels, promoting osteoblast proliferation and differentiation (Abed and Moreau, 2007; 2009). TRPM7 regulates intracellular Ca^2+^/Mg^2+^ balance and influences expression of osteogenic markers such as alkaline phosphatase and osteocalcin.

Magnesium deficiency reduces serum alkaline phosphatase and osteocalcin levels, impairing bone growth and development. TRPM7 silencing inhibits cadmium-induced osteoporosis under magnesium starvation (Martineau, et al., 2010), suggesting that TRPM7 promotes cadmium absorption during magnesium depletion (Jimenez, et al., 2020). It was reported that magnesium ions contribute to the upregulated expression of TRPM7, while the TRPM7 induces the influx of magnesium ions in cells. Also, it was reported that magnesium perform a bidirectional effect on osteogenic differentiation by regulating immune microenvironment in different stages by TRPM7. In the early inflammatory phase, Mg^2+^ upregulates TRPM7 in the monocyte-macrophage lineage, initiating TRPM7 kinase-mediated signaling that promotes inflammatory cytokine expression and builds a pro-osteogenic immune microenvironment. However, sustained Mg^2+^ exposure in the later remodeling phase causes overactive NF-κB signaling in macrophages, more osteoclastic-like cells, suppressed hydroxyapatite precipitation and delayed bone maturation, with these negative effects overriding the initial pro-osteogenic benefits of Mg^2+^ (Qiao, et al., 2021). Overall, TRPM7 plays a critical role in bone metabolism, and abnormal magnesium concentrations affect bone metabolism via TRPM7.

### 3.4 Wnt signaling network

Wnt network comprises the canonical Wnt/β-catenin pathway and non-canonical Wnt pathways (the Wnt/Ca^2+^ pathway and the PCP pathway) (Pandur, et al., 2002). The canonical Wnt pathway activates nuclear gene transcription through β-catenin. When extracellular Wnt ligands bind to the membrane receptor Frizzled, a series of interactions among membrane and cytoplasmic proteins leads to dimer formation, allowing β-catenin to accumulate in the cytoplasm. β-Catenin then translocates into the nucleus, where it associates with T-cell factor (TCF)/lymphoid enhancer factor (LEF) to form a transcriptional complex that activates downstream target genes and promotes osteoblast differentiation and proliferation (Mulholland, et al., 2005).

The Wnt/β-catenin pathway is a key signaling cascade in osteocyte differentiation and proliferation, and its activity is closely related to bone mass (Maruyama, et al., 2007). In addition, β-catenin can induce mesenchymal stem cells to differentiate into osteoblasts by enhancing their responsiveness to bone morphogenetic protein-2 (BMP-2). This suggests that β-catenin provides a molecular inductive signal during the proliferation and differentiation of osteoprogenitor cells and osteoblasts, and is regulated by BMP-2. These findings indicate that BMPs and the canonical Wnt pathway cooperate to regulate osteoblast differentiation. Moreover, BMP-2 can also act through its receptor BMPR2 to activate PI3K/Akt and β-catenin signaling, thereby inhibiting osteoblast apoptosis (Wang and Guo, 2013).

Wnt proteins can regulate osteoblast proliferation and survival by activating the Src/ERK and PI3K/Akt pathways (Dufour, et al., 2008), further suggesting crosstalk among BMP-2, the PI3K/Akt pathway, and the canonical Wnt pathway during osteoblast differentiation and bone formation. Since the PI3K/Akt pathway involved in bone metabolism is influenced by magnesium ion concentrations, it is reasonable to speculate whether the Wnt pathway in bone metabolism is likewise affected by magnesium levels; however, the regulatory mechanism of magnesium ions on Wnt signaling remains unclear.

## 4 Magnesium related bone metabolic diseases

Magnesium intake and absorption are dynamic processes. Decreased magnesium levels can lead to the release of inflammatory factors (Weglicki, 2012), causing a series of related diseases such as migraine, type II diabetes, metabolic syndrome, hypertension, atherosclerosis, cardiogenic sudden death, and even colon cancer (Rosanoff, et al., 2012). In recent years, studies have found that osteoporosis is also related to the decrease of magnesium. Osteoporosis is a bone disease related to abnormal bone metabolism throughout the body, mainly manifested as changes in the microstructure of bone tissue, a decreasing number of bone trabeculae, a reduction in bone mass, and an increase in the brittleness of bone tissue. Magnesium not only directly affects the function of bone cells and the growth of hydroxyapatite crystals, but also can affect the stability of bone tissue by regulating related factors in the bone metabolism process. Therefore, the decrease of magnesium can directly affect bone density. However, there are different explanations for the mechanism by which magnesium deficiency causes a reduction in bone mass. Current research suggests that magnesium can affect the division and growth of bone cells by regulating factors related to bone metabolism, so its deficiency may cause a decrease in bone formation. In addition, low magnesium can disrupt the phosphatidylinositol system and reduce the activity of adenylate cyclase, leading to the anti-parathyroid hormone (PTH) effect in the bone and kidneys, and by increasing magnesium, the PTH response of the body can be restored, thereby affecting the strength of the bone. In addition to the disturbance of normal bone metabolism caused by the decrease of magnesium, the increase in the concentration of magnesium ions also affects the physiological activities of bones. *In vitro* experiments have found that high magnesium significantly inhibits the deposition of mineral matrix in osteoblasts and reduces the activity of alkaline phosphatase, a differentiation marker of osteoblasts. In addition, high concentrations of magnesium can compete with transport proteins to change the concentrations of various cations including calcium ions inside the cells, thereby affecting the metabolism of bone cells. When the crystalline deposition of minerals in bone tissue is abnormal, it will lead to a decrease in the activity of osteoblasts, a widening of the growth plate, and a shortening and thickening of the bones, resulting in a softening-like manifestation of the bones (Riond, et al., 2000). Animal experiments have found that excessive magnesium stimulates bone resorption, but there are also reports showing that high magnesium inhibits the proliferation and adhesion of osteoblasts and that the mineralization level of cells decreases significantly with the increase in magnesium concentration (Yun, et al., 2010). Therefore, abnormal level of magnesium can affect the stability of bone tissue.

## 5 Clinical application of magnesium alloys in bone tissue

Magnesium alloys are a research hotspot in biomaterials due to their excellent biocompatibility, low density, and high strength-to-weight ratio, making them ideal biomimetic bone implant materials (Brar, et al., 2013). Their elastic modulus is close to natural bone, reducing mechanical stress shielding and improving implant stability (Chanlalit, et al., 2012; Ghosh, et al., 2013; Nagels, et al., 2003; Schmidutz, et al., 2014). As biodegradable metals, magnesium alloys degrade completely *in vivo* and have attracted increasing attention for hard tissue implants. Their degradation eliminates the need for secondary surgery, reducing clinical and economic burdens. More important, magnesium alloys exhibit osteoinductive properties, promoting bone growth during degradation. The study established a fracture model of the ulna in New Zealand white rabbits. A magnesium alloy (ZK60) internal fixation system was used for stability, and it was confirmed that magnesium alloy is an ideal new type of internal fixation material (Kraus, et al., 2012) (Table 2).

**TABLE 2:** The advantages and challenges of magnesium alloys as orthopedic implant materials.

Property	Core advantages	Challenges
Biocompatibility	Biodegradable (no residues); Mg^2+^ degradation product participates in bone metabolism	Difficult to precisely control local degradation rate
Mechanical performance	Elastic modulus close to natural bone (reduced stress shielding); high strength-to-weight ratio	Mechanical performance decline with degradation
Osteoinductivity	Released Mg^2+^ promotes local bone formation/osseointegration; increased peri-implant mineralization	Hydrogen gas formation and potential local tissue compatibility issues
Clinical application	Eliminates secondary removal surgery; suitable for fracture fixation/bone defect repair	Lack of standardized guidelines; insufficient large-sample clinical evidence

We list the advantages and key unsolved challenges of the magnesium alloys as orthopedic implant materials in this table.

Magnesium ions, the fourth most abundant cation in the body, stabilize DNA and RNA and regulate multiple metabolic processes, including bone metabolism (Hartwig, 2001). Magnesium ions constitute 0.5%–1% of bone mineral content and regulate bone metabolism via hormones, growth factors, and signaling pathways, as well as direct effects on bone tissue (D'Amelio, et al., 2009; Nomura, et al., 2001; Pramojanee, et al., 2014). Studies show increased mineralization and bone mass around magnesium implants (Witte, et al., 2006). Degradation products promote bone healing and osseointegration (Castellani, et al., 2011; Staiger, et al., 2006; Witte, et al., 2005), while excess magnesium is excreted by the kidneys, maintaining safe serum levels (Saris, et al., 2000; Vormann, 2003). Therefore, elucidating the molecular mechanisms of magnesium ions in bone metabolism is critical for guiding clinical applications of magnesium alloys.

In terms of clinical translation, a growing number of clinical trials have confirmed the safety and efficacy of magnesium alloy implants in orthopedic practice. In a 3 years results of a randomized clinical trial (RCT) including 26 patients with symptomatic hallux valgus, bioabsorbable magnesium implants showed comparable clinical results to titanium standard implants after distal modified metatarsal osteotomy and were more suitable for radiologic analysis (Plaass, et al., 2018). Also, an RCT study on Mg-containing composite porous scaffold for bone defect repair has strong potential for future clinical applications where it might synergistically facilitate bone regeneration and early healing in critical-size bone defects (Gong, et al., 2026). There are many similar studies. A meta-analysis of 3 RCT, 1 retrospective study, 2 case-control studies, and 2 prospective studies including 468 patients (230 Mg screws and 213 Titanium screws) showed that the application of Mg screws in orthopedic surgery is equivalent to standard metal fixation devices (Sukotjo, et al., 2020).

Despite the broad application prospects, magnesium alloy implants still have the following limitations and challenges in clinical orthopedic application. The first is the uncontrollable degradation rate. The degradation rate of most magnesium alloys *in vivo* is too fast, which may lead to the loss of mechanical support before the completion of fracture healing, and the local rapid degradation will cause excessive accumulation of hydrogen gas, leading to subcutaneous emphysema, soft tissue swelling and other adverse reactions (Li Y. et al., 2019). Local magnesium ion concentration control is another problem. Excessive local magnesium ion concentration caused by rapid degradation will inhibit osteoblast mineralization and even lead to local inflammatory reaction. Zheng et al. tried to solve this problem in the treatment of osteoarthritis (Zheng, et al., 2024), while how to precisely deliver appropriate magnesium in bone tissues is still need to be explored. In the clinical practice, we find that the lack of clinical data is the major limitation, while the long-term safety of magnesium alloy implants in the human body (over 5 years) is still unknown. Also, the safety of magnesium alloy implants in patients with renal insufficiency (impaired magnesium excretion) and pediatric patients with skeletal development is still lack of sufficient clinical evidence.

## 6 Magnesium nursing management in orthopedic patients

### 6.1 Magnesium supplementation strategies

Comprehensive assessment of magnesium status is essential for identifying the degree of deficiency and guiding supplementation strategies. Although serum magnesium measurement is readily available, it reflects only approximately 1% of total body magnesium and may not detect intracellular deficiency (Dominguez, et al., 2020). Nurses should employ additional assessment methods, including dietary history and symptom evaluation (e.g., muscle weakness, cramps, and fatigue) (Capozzi, et al., 2020). A comprehensive magnesium assessment should be conducted for orthopedic patients at risk of magnesium deficiency, including those with prolonged immobilization, chronic pain, or those receiving medications that affect magnesium metabolism (e.g., diuretics and proton pump inhibitors) (da Silva, et al., 2020).

Dietary intake is the primary source of magnesium and should be considered the first-line intervention (Groenendijk, et al., 2022; Kleczkowski and Igamberdiev, 2023). Magnesium-rich foods include vegetables, nuts, whole grains, legumes, and fish. Nurses should provide dietary counseling and develop interactive nutrition supplementation plans through patient education initiatives. The recommended dietary allowance (RDA) for magnesium is 310–320 mg/day for adult women and 400–420 mg/day for adult men, with increased requirements in older adults and patients recovering from orthopedic diseases. However, many orthopedic patients cannot meet the RDA through diet alone and therefore require nutritional supplementation (Bruna, et al., 2022). Nurses should educate patients about dietary sources and collaborate with registered dietitians to optimize nutritional intake (Table 3).

**TABLE 3:** Magnesium supplementation for orthopedic patients.

Supplementation purpose	Indicated populations	Recommended dose	Preferred form	Key precautions
Deficiency prevention	Postoperative patients, prolonged immobilization	200–400 mg/day, divided doses	Dietary (green vegetables, nuts, grains); Mg glycinate/malate	Diet as first-line intervention
Deficiency treatment	Hypomagnesemia, muscle cramp/weakness	400–800 mg/day, divided doses	Oral Mg glycinate/malate (high bioavailability)	Avoid sole Mg oxide; monitor gastrointestinal side effects
Perioperative optimization	Elective orthopedic surgery patients	Preop: 2–4 weeks basic supplement Intraop: 20–40 mg/kg Postop: 400–600 mg/day	Preop oral + intraop IV + postop oral	Monitor serum Mg intraop; adjust by recovery status postop

All doses refer to elemental magnesium. Supplementation should be individualized according to clinical status, renal function, and baseline magnesium levels. Serum magnesium monitoring is recommended, particularly in perioperative settings, to minimize the risk of hypermagnesemia. Organic magnesium salts (e.g., glycinate and malate) are preferred due to their higher bioavailability and improved gastrointestinal tolerance compared with inorganic forms such as magnesium oxide. Intravenous magnesium administration should be performed under appropriate clinical supervision.

When dietary intake is insufficient, pharmacological magnesium supplementation becomes necessary. Various magnesium formulations are available, differing in bioavailability and gastrointestinal tolerability (Long, et al., 2022). Magnesium glycinate and magnesium malate exhibit excellent absorption and tolerability, making them suitable for oral supplementation in orthopedic patients. Magnesium oxide is inexpensive but has poor bioavailability and frequently causes gastrointestinal side effects (Veronese, et al., 2022).

The recommended supplementation dosage is 200–400 mg/day for prevention and 400–800 mg/day for treatment, administered in divided doses to enhance absorption (Serefko, et al., 2016). Nurses should monitor gastrointestinal side effects (e.g., diarrhea and nausea) and adjust the dosage accordingly. Supplementation regimens should be individualized based on baseline magnesium levels, dietary intake, and clinical response (Costello, et al., 2016).

It should be emphasized that magnesium does not regulate bone metabolism alone, but has a significant synergistic effect with calcium, vitamin D, and vitamin K2, which are key nutrients for bone health. Magnesium is a key cofactor for calcium absorption and metabolism, which can promote the deposition of calcium in bone tissue rather than soft tissue, reduce the risk of vascular calcification caused by simple calcium supplementation, and the ratio of dietary calcium to magnesium intake is recommended to be controlled at 2:1 to 3:1. Vitamin D is an important regulator of intestinal magnesium absorption, and vitamin D deficiency will significantly reduce the absorption rate of magnesium; at the same time, magnesium is a necessary cofactor for the hydroxylation activation of vitamin D in the liver and kidney, and magnesium deficiency will lead to the inactivation of vitamin D, forming a vicious circle. Magnesium can upregulate the expression of osteocalcin, and vitamin K2 can carboxylate and activate osteocalcin, which together promote the mineralization of bone matrix and inhibits bone resorption.

### 6.2 Perioperative management of magnesium

Intraoperative magnesium management is crucial for optimizing surgical outcomes in orthopedic patients. Preoperative evaluation should identify magnesium deficiency, and supplementation should begin 2–4 weeks before elective surgery to optimize tissue magnesium levels (Shin, et al., 2020). Intraoperative magnesium administration (20–40 mg/kg) can reduce anesthetic requirements, maintain hemodynamic stability, and alleviate postoperative pain (Hung, et al., 2024). Postoperatively, magnesium supplementation (400–600 mg/day) can accelerate pain relief, reduce inflammation, and promote functional recovery (Choi, et al., 2021). Nurses should collaborate with anesthesiologists and surgeons to monitor serum magnesium levels in high-risk patients and adjust supplementation based on clinical response.

### 6.3 Interactions between magnesium and other medications

Many medications commonly prescribed for orthopedic patients can interact with magnesium, thereby affecting its absorption efficiency (Table 4). Proton pump inhibitors (PPIs) and H_2_ receptor antagonists reduce gastric acid secretion, which may impair magnesium absorption and increase the risk of hypomagnesemia (Durlach, et al., 1994). Bisphosphonates should be administered at least 2 h apart from magnesium supplements to prevent chelation reactions and reduced drug efficacy (Reddy and Edwards, 2019). Diuretics, particularly loop diuretics and thiazide diuretics, increase urinary magnesium excretion; therefore, magnesium supplementation is recommended for patients requiring long-term diuretic therapy. Aminoglycosides, amphotericin B, and cisplatin can induce renal magnesium wasting, necessitating monitoring and supplementation (van Orten-Luiten, et al., 2019). Nurses should review patients’ medication lists to identify potential interactions and coordinate magnesium supplementation timing with drug administration schedules.

**TABLE 4:** Drug-magnesium interactions and nursing interventions in orthopedics.

Drugs	Representative drugs	Interaction with magnesium	Nursing interventions
Proton pump inhibitors	Omeprazole, lansoprazole	Reduced gastric acid → impaired Mg absorption → hypomagnesemia risk	Regular Mg status assessment; prioritize dietary Mg supplement
Bisphosphonates	Alendronate, zoledronic acid	Chelation reaction → reduced bioavailability of both	Administer with ≥2 h interval
Diuretics	Furosemide, hydrochlorothiazide	Increased urinary Mg excretion → Mg loss	Routine Mg supplement; monitor serum Mg and adjust dose
Aminoglycosides	Gentamicin, amikacin	Renal Mg wasting → decreased serum Mg	Monitor serum/urinary mg; prompt supplement if deficient

Drug–magnesium interactions may affect absorption and excretion. Monitoring of serum Mg is recommended, especially with proton pump inhibitors, diuretics, and aminoglycosides. Dosing intervals should be maintained for chelating drugs (e.g., bisphosphonates). Interventions should be individualized based on renal function and electrolyte status.

### 6.4 Nursing care for magnesium deficiency symptoms

Magnesium deficiency can cause symptoms such as muscle cramps, weakness, fatigue, and pain—symptoms commonly observed in orthopedic patients (Cao, et al., 2018). Nurses should identify these symptoms and implement targeted interventions, including magnesium supplementation, stretching exercises, thermotherapy, and massage. Muscle cramps, particularly nocturnal leg cramps commonly experienced by orthopedic patients, respond well to magnesium supplementation (Chiu, et al., 2016). Nurses should educate patients on preventive strategies, including adequate magnesium intake, appropriate stretching, and sufficient hydration. For patients with postoperative pain, magnesium supplementation can serve as an adjunct to pharmacological pain management and may reduce the need for opioid analgesics.

### 6.5 Nursing management of hypermagnesemia in orthopedic patients

Standardized nursing management of hypermagnesemia is an indispensable part of the whole-cycle care for orthopedic patients, which runs through preoperative assessment, perioperative monitoring, postoperative follow-up and long-term home care. The core nursing objectives include early identification of high-risk populations, prevention of excessive magnesium exposure, timely intervention of confirmed hypermagnesemia, and reduction of adverse clinical outcomes.

Nurses should conduct routine hypermagnesemia risk screening for all orthopedic patients at admission, and dynamic reassessment during hospitalization and follow-up. The core high-risk factors include: impaired renal function (estimated glomerular filtration rate <60 mL/min/1.73 m^2^), long-term use of medications that inhibit magnesium excretion (including potassium-sparing diuretics, angiotensin-converting enzyme inhibitors, and angiotensin receptor blockers), excessive oral magnesium supplementation, implantation of magnesium-based biodegradable implants, diabetes mellitus, advanced age, and tumor-related bone diseases. For high-risk patients, comprehensive magnesium status assessment should be implemented, combining serum magnesium testing (the core monitoring indicator), dietary history review, medication reconciliation, and clinical symptom evaluation. For patients with magnesium-based implants, in addition to regular serum magnesium monitoring, nurses should also pay close attention to local symptoms around the implant, including swelling, subcutaneous emphysema, pain, and wound exudation, to identify local excessive magnesium exposure early. Prevention is the core of hypermagnesemia management in orthopedic patients, and the key is to standardize magnesium intervention and avoid excessive exposure. First, nurses should adhere to the principle of “diet first, individualized supplementation”, strictly implement the recommended magnesium supplementation dosage in Table 3, and avoid blind high-dose magnesium supplementation. For patients with normal renal function, the maximum daily oral elemental magnesium dose should not exceed 800 mg, and for patients with renal impairment, prophylactic magnesium supplementation should be avoided unless clear hypomagnesemia is confirmed. Second, nurses should standardize the management of drug-magnesium interactions. For patients taking medications that inhibit magnesium excretion, regular serum magnesium monitoring should be performed every 1–2 weeks, and unnecessary magnesium supplementation should be avoided. For patients with magnesium-based implants, nurses should provide targeted health education, inform patients of the signs of abnormal implant degradation, and arrange regular follow-up to monitor implant degradation and local tissue response. Third, dietary guidance should be provided for high-risk patients. For CKD stage 3–5 patients, nurses should collaborate with dietitians to formulate a low-magnesium diet plan, guide patients to avoid high-magnesium foods including nuts, whole grains, legumes and green leafy vegetables, and avoid magnesium-containing over-the-counter drugs such as antacids and laxatives.

Nurses should provide targeted health education for high-risk patients and their caregivers, including the hazards of excessive magnesium intake, the correct usage of magnesium supplements, the need for regular serum magnesium monitoring, and the early warning signs of hypermagnesemia (muscle weakness, numbness, nausea, dizziness, etc.). For patients discharged with oral magnesium supplements, nurses should emphasize that they must take the medication strictly according to the prescribed dose and avoid self-increasing the dose or combining multiple magnesium-containing preparations. For patients with magnesium-based implants, patients should be informed to seek medical attention immediately if local swelling, pain, or abnormal skin changes occur around the implant.

### 6.6 Magnesium nursing management in special orthopedic populations

The magnesium metabolism characteristics and nutritional requirements of special orthopedic populations are significantly different from those of general adult patients. Nurses should formulate individualized magnesium management strategies based on the physiological characteristics and comorbidities of different populations, to improve the safety and effectiveness of intervention.

The elderly patients are of the high-risk of magnesium deficiency, due to reduced intestinal magnesium absorption efficiency, decreased dietary intake, multiple comorbidities, and long-term use of multiple drugs (such as diuretics, proton pump inhibitors). Meanwhile, elderly patients often have osteoporosis, high fracture risk, and poor postoperative recovery ability, which are closely related to long-term subclinical magnesium deficiency. So, it is worth highlighting that routine magnesium status assessment for all elderly orthopedic patients is necessary, including dietary history, medication history, clinical symptom assessment and serum magnesium detection. Also, the elderly might be benefit from dietary magnesium supplementation and formulate easy-to-execute dietary plans according to the chewing and digestive function of the elderly. Also, it is optional for prophylactic supplementation of magnesium based on the serum concentration of Mg and the renal function in different patients, especially for those with long-term use of drugs that affect magnesium metabolism.

Pediatric patients are in the critical stage of skeletal growth and development, with high magnesium requirements for bone formation and growth. The RDA of magnesium for children is 30–80 mg/day for infants aged 0–6 months, 80–130 mg/day for infants aged 6–12 months, 130–240 mg/day for children aged 1–3 years, 240–350 mg/day for children aged 4–8 years, 350–410 mg/day for children aged 9–13 years, and 360–420 mg/day for adolescents aged 14–18 years (male 420 mg/day, female 360 mg/day). So, based on the high demand of magnesium in pediatric patients, the dietary magnesium intake from natural foods to meet the needs of growth and development is important. Also, for those patients with fracture delayed healing, skeletal dysplasia or long-term use of glucocorticoids, it is important to assess the status of magnesium. Pharmacological supplementation should be carried out under the guidance of pediatricians and dietitians, strictly control the dose according to age and body weight, and avoid excessive magnesium intake affecting skeletal development.

Pregnancy is a special physiological stage with significantly increased magnesium requirements, while the RDA of magnesium for pregnant women is 350–400 mg/day, which is 10%–25% higher than that of non-pregnant women. Magnesium is not only related to the skeletal health of pregnant women, but also essential for the skeletal development of the fetus. Meanwhile, magnesium supplementation can reduce the risk of pregnancy-induced hypertension, premature uterine contraction and muscle cramps in pregnant women. So, it is important to strengthen dietary guidance for pregnant women, encourage the intake of magnesium-rich foods that are easy to digest and absorb. Also, the assessment of magnesium status during prenatal examination is important and pharmacological supplementation should be strictly in accordance with the guidance of obstetricians. The daily dose should not exceed 400 mg/day and serum magnesium level should be monitored to avoid adverse effects on mother and fetus.

Because magnesium is excreted from the body by the kidneys, the concentration of magnesium in those patients with chronic kidney disease and diabetes mellitus should be paid additional attention. Hyperglycemia will increase urinary magnesium excretion, and more than 30% of diabetic patients have hypomagnesemia. Magnesium deficiency will further aggravate insulin resistance, affect bone metabolism, and increase the risk of diabetic osteoporosis and delayed healing fracture. So, it is worth noting that regular monitoring of serum magnesium and blood glucose should be paid attention in diabetes mellitus patients. The kidney is the main organ of magnesium excretion, and CKD patients have impaired magnesium excretion capacity, which is prone to hypermagnesemia. Nursing strategies include that the strictly limit magnesium supplementation for patients with CKD stage 3–5, regularly monitor serum magnesium level, avoid magnesium-rich foods and magnesium-containing drugs, and formulate individualized magnesium management plans in collaboration with nephrologists.

## 7 Discussion

Magnesium has emerged as a multifunctional regulator of skeletal homeostasis that integrates molecular signaling, biomaterial science, and clinical management. Consistent with the scope defined in the introduction, this review focuses on the functional regulatory interplay between magnesium and key bone metabolic pathways, rather than an isolated description of the canonical signaling cascades of these pathways themselves. This review highlights that magnesium is not merely a structural component of bone mineral but a dynamic bioactive ion capable of coordinating osteoblast activity, osteoclast differentiation, and stem cell fate decisions through multiple interconnected signaling pathways. Among these, the PI3K/Akt pathway appears to function as a central signaling hub, mediating magnesium-dependent effects on osteoblast proliferation, apoptosis resistance, and mesenchymal stem cell osteogenic commitment.

The OPG/RANKL/RANK axis further illustrates the systemic regulatory role of magnesium in bone resorption. Magnesium deficiency shifts the balance toward increased RANKL signaling and osteoclast activation, providing a mechanistic explanation for the association between hypomagnesemia and osteoporosis observed in epidemiological and experimental studies. Meanwhile, magnesium-sensitive ion channels such as TRPM6 and TRPM7 represent a more direct sensing mechanism by which bone cells detect extracellular magnesium fluctuations and translate them into intracellular calcium–magnesium signaling responses that regulate osteogenic gene expression. These pathways not only play important roles in orthopedic diseases, but also in a lot of diseases, which indicates that magnesium might contribute to the treatment for these diseases (Cheng, et al., 2025; Huang J. et al., 2024; Liu, et al., 2023; Nie, et al., 2023; Qin, et al., 2024). Together, these pathways suggest that magnesium participates in bone metabolism through both transcriptional regulation and ion-channel-mediated cellular signaling. Also, the elements of the same group such as strontium and calcium are also in need to be explored in treating orthopedic diseases such as osteoporosis (Huang L. et al., 2024; Pan, et al., 2022).

Although emerging evidence suggests potential interactions between magnesium signaling and the Wnt/β-catenin network, the precise regulatory mechanisms remain insufficiently characterized. Given the established crosstalk among Wnt signaling, BMP activity, and PI3K/Akt pathways in osteogenesis, it is plausible that magnesium may influence bone formation partly through modulation of this signaling network. Future studies integrating transcriptomic, proteomic, and ion-flux analyses will be essential to clarify whether magnesium directly regulates Wnt signaling or acts indirectly through upstream metabolic or kinase-dependent pathways.

From a translational perspective, magnesium-based biodegradable implants represent one of the most promising clinical applications of magnesium biology. Their elastic modulus closely matches that of natural bone, minimizing stress shielding while simultaneously releasing bioactive magnesium ions that may enhance local osteogenesis and fracture healing. Unlike permanent metallic implants, magnesium alloys degrade *in vivo*, potentially eliminating the need for secondary removal surgery. However, challenges remain regarding degradation rate control, hydrogen gas formation, and local ion concentration management, all of which require continued optimization in materials engineering and clinical monitoring.

Equally important, this review emphasizes that effective magnesium management in orthopedic patients extends beyond biomaterials into perioperative care and long-term nutritional strategies. Since serum magnesium reflects only a small fraction of total body stores, comprehensive assessment combining dietary history, medication review, and clinical symptom evaluation may provide a more accurate evaluation of magnesium status. Integrating magnesium monitoring into orthopedic nursing protocols may therefore improve postoperative recovery, pain control, and functional outcomes.

From the perspective of clinical nursing practice, magnesium management has become an important part of the whole-cycle care of orthopedic patients. This review systematically clarifies that magnesium intervention runs through the whole process of orthopedic patient care: preoperative optimization of magnesium nutritional status can improve the tolerance of patients to surgery and reduce the risk of postoperative complications; intraoperative magnesium administration can maintain hemodynamic stability and reduce the dosage of anesthetics and analgesics; postoperative magnesium supplementation can alleviate pain, reduce inflammatory response, promote fracture healing and functional recovery; long-term magnesium management can improve bone mineral density, reduce the risk of osteoporosis and re-fracture, and improve the long-term prognosis of orthopedic patients. However, the current clinical application of magnesium in orthopedic nursing still faces many challenges. First, most orthopedic nurses lack systematic understanding of magnesium’s role in bone metabolism and clinical nursing management, and there is no standardized nursing operation process. Second, the current assessment method of magnesium status is still limited, serum magnesium cannot accurately reflect the total magnesium level in the body, and there is a lack of simple and effective bedside assessment tools for clinical nursing. Also, there is no unified clinical nursing guideline for magnesium management in different orthopedic disease populations and special populations, and the existing evidence is mostly derived from basic research and clinical drug trials, with few high-quality nursing intervention studies.

Overall, Magnesium holds significant promise in orthopedic research and clinical practice, particularly in personalized supplementation strategies and the development of biodegradable magnesium-based implants. Future advances in biomarker development and biomaterial engineering may enable precise regulation of magnesium homeostasis and controlled implant degradation. However, several challenges remain, including the limited accuracy of serum magnesium measurements, the lack of standardized clinical guidelines for supplementation, the dose-dependent dual effects of magnesium on bone cells, and the rapid corrosion and gas formation associated with magnesium alloys. Furthermore, most current evidence is derived from preclinical studies, and large-scale clinical trials are required to validate safety and efficacy. Addressing these challenges will be critical for translating magnesium-based therapies and biomaterials into routine orthopedic clinical practice.
